# Investment analysis of the private firm under different financial arrangements in infrastructure projects

**DOI:** 10.1371/journal.pone.0287418

**Published:** 2024-02-16

**Authors:** Qianxing Ding, Shanshan Huang, Guohua Fu, Bing Wang

**Affiliations:** 1 College of Management and Economics, Tianjin University, Tianjin, PR China; 2 School of Management, Hainan University, Haikou, PR China; 3 School of Management, Zhejiang University of Technology, Hangzhou, PR China; Universiti Malaysia Sabah, MALAYSIA

## Abstract

This study investigates the impact of various financial arrangements on the investment behavior of the private firm in PPP (Public-Private Partnership) projects. The results manifest that: first, the private firm will invest in the project earlier under long-term debt financing than under short-term debt financing or all equity financing; second, the investment boundary of the private sector decreasing with the probability of obtaining long-term debt financing under short-term debt financing, while increasing with the probability of obtaining long-term debt financing under long-term debt financing; third, the optimal debt level under short-term debt financing displays a U-shaped relationship with the refinancing risk probability; fourth, under short-term debt financing, the difference in the optimal capital structure between projects with different volatility of cash flow is larger when the refinancing risk probability is lower; and fifth, the private firm may exit the project earlier under short-term debt financing than under long-term debt financing. These results can help us to understand the investment behavior of the private firm under different financial arrangements.

## Introduction

With the financial burden of governments all over the world increasing, it is increasingly harder to meet the requirement of infrastructure development with the government’s financial budget alone [1, 2]. Because of this, attracting the private firm to invest in infrastructure development has become a hot topic [3, 4]. Several new project delivery methods have been proposed, *e.g*. the Build-Operate-Transfer (BOT) model, the Build-Own-Operate-Transfer (BOOT) model and the Design-Build-Finance-Operate (DBFO) model, in which the private firm is responsible for the construction and financing of the project, and recovers investment by operating the project up to 20–30 years [5, 6]. Since these project delivery methods can help reduce the financial burden of the government and improve the operating efficiency of the infrastructure, they are widely used in many countries, *e.g*. the US, the UK, Canada and China [7–9]. In practice, due to the billions of capital required of developing infrastructure, *e.g*. a toll highway project, the private firm has to raise a large amount of debt capitals. In the US, TIFIA (Transportation Infrastructure Finance and Innovation Act) loan, a kind of long-term and low-cost federal loan, has been widely used by the government to attract the private entities to invest in PPP projects. Here PPP is regarded as a general term covering all contracted relationships between the public and private sectors to produce public infrastructure via private financing, including transport, water, electrical power, telecommunications, and sport facilities’ projects. Since the program inception, TIFIA has approved 81 loans totaling $-30.8 billion to stimulate over $-111 billion of transportation infrastructure investments in 22 states. The long-term project finance that can cover the operating period of the project is preferred by the private firm. Nonetheless, after the 2008 financial crisis, the international bank organization is more prudent to arrange long-term debt financing [10]. Therefore, it is more difficult for the private firm to raise long-term debt financing. In order to obtain enough capitals to develop the infrastructure, short-term debt financing is an alternative method, but the private firm needs to shoulder the refinancing risk [11]. Previous studies have indicated that the financing arrangement can influence the investment decision of the private firm [12–14]; this study further investigates the investment decision of the private firm under long-term debt financing and short-term debt financing respectively. Especially, the three investment decisions of the private firm focused on are: the investment boundary, the optimal capital structure and the default boundary.

Since the seminal study of [15], plenty of studies have been conducted to investigate the interaction between the firm’s investment and financing decisions by considering various market frictions, e.g. product market competition [16, 17], asymmetric information [18, 19], financing frictions [20–22] and other factors [12, 14, 23]. However, most of these studies assumed that the firm is financed by a single type debt. As highlighted in the study of [21], the shortcoming of considering corporate debt as uniform is that different financing arrangements have quite different effects on the investment strategies of the firm. [12, 21] differentiated the corporate debt as bank debt and market debt (bonds) and investigated the investment strategy of the firm under two kinds of corporate debt. Nonetheless, a major limitation that still exists is that previous studies took the corporate debt of the firm as the long-term debt by default, and different types of debt structure may also have a greatly influence on the investment strategies of the firm.

In this study, it is assumed that the firm can raise two types of debt structure: long-term debt financing and short-term debt financing. An obvious difference between two types of debt structure is that the firm should shoulder the refinancing risk under short-term debt financing. Besides, following the study of [22], this study considers the financing friction having existed in the credit market. That is, the firm needs to take cost to search the debt financing, but may not obtain the debt financing definitely, which captures the deterioration of the global financing market. Specifically, the firm needs to take more cost to obtain the long-term debt loan since the bank is more careful to arrange long-term debt financing after the 2008 financial crisis. By investigating how financing friction along with these two types of debt financing influences the investment strategies of the firm, this study can explain the necessity of providing TIFIA loan for the PPP project and the reason for difference in capital structure among different PPP projects.

The remainder of this paper is organized as follows. The next section (the second section) describes the basic model. The third section investigates the investment strategies of the firm under different financial arrangements. The fourth section discusses the model implication by means of some numerical analyses. The final section makes a conclusion of this study.

## The modeling

When participating in the infrastructure project, private firms usually set up a new joint venture company (project company) to build and operate the project, which can separate the project risk from the original asset of private firms [7, 9]. In other words, there is no asset in place for the project company before the private firm invests in the project. Since the cash flow of the infrastructure project is hard to predict, this study follows the traditional assumption that the cash flow of the project (*X*_*t*_) follows the standard Wiener process [3, 4], namely, *dX*_*t*_ = *μX*_*t*_*dt* + *σX*_*t*_*dW*_*t*_, where *μ* means the expected rate of return of the project, and satisfies *μ* < *r*, *r* means the risk-free interest rate, and *σ* means the volatility of cash flow.

The private firm needs to raise debt capitals for the construction cost of the project, and two alternative ways are available. One way is to obtain long-term debt financing that can cover the total operating period of the project, the other way is to obtain short-term debt financing first and then conduct the refinancing. However, due to the tightened financial market after the financial crisis of 2008, the private firm needs to take longer and spend more to search the long-term debt financing in the financial market. Suppose the search cost for the long-term debt is *ϕ*_1_, the search cost for the short-term debt is *ϕ*_2_, and the magnitude of them satisfies *ϕ*_2_ < *ϕ*_1_. Besides, suppose the probability of obtaining long-term debt financing is *δ*_1_, the probability of obtaining short-term debt financing is *δ*_2_, and the magnitude of them satisfies *δ*_1_ < *δ*_2_. To make sure the financing arrangement is feasible, the probability of obtaining debt financing should not be too small (*δ*_*i*_ ≥ (1 + *r*)/2, *i* = 1, 2).

At first sight, the private firm needs to take more cost to search long-term debt financing, but has a lower probability of obtaining the financing. Therefore, the private firm has reason to abandon this financing option and dedicate itself to raising short-term debt financing. Nonetheless, short-term debt financing will make the private firm shoulder the refinancing risk, which may be costly for the private firm. Since the investment is irreversible, once the refinancing risk occurs, the private firm will lose their previous investment. Suppose the probability of refinancing risk is *ρ*, and the value of *ρ* depends on the condition of financial market and the profitability of project when the private firm needs to roll over the current debt. Suppose *ρ* = (1 − *aδ*_1_)(1 − *aδ*_2_), where *a* captures the improved (deteriorated) financial market or better (worse) project profitability if *a* ≥ 1 (*a* < 1). Therefore, to make a better financing decision, the private firm should trade off the scarceness of long-term debt and the refinancing risk of short-term debt. Suppose the financial structure of the project company is stable, or the private firm raises the same amount of debt to cover the original debt during the refinancing process. This study considers the debt contract to be characterized by a perpetual flow of coupon payments. Suppose coupon payments are *C*^*L*^ under long-term debt financing and *C*^*S*^ under short-term debt financing, where the superscripts “L” and “S” stand for the Long-term and the Short-term, respectively. Finally, suppose the tax rate is *τ*, and the construction cost is *I*. The notations and their explanations used in this study are presented in the Table 1.

**Table 1:** The notations and explanations.

*μ*	The expected rate of return of the project
*σ*	The volatility of cash flow
*r*	The risk-free interest rate
*τ*	The tax rate
*α*	The recovery rate of the project in default
*ϕ*_1_	The search cost for the long-term debt
*δ*_1_	The probability of accessing to long-term debt
*ϕ*_2_	The search cost for the short-term debt
*δ*_2_	The probability of accessing to short-term debt
*ρ*	The refinancing risk probability
*a*	The condition of the project profitability
*I*	The construction cost
*C* ^i*^	The optimal debt level under different financing (*i* = *L*, *S*)
$X_{B}^{i}$	The default boundary under different financing (*i* = *L*, *S*)
$X_{I}^{i}$	The investment boundary under different financing (*i* = *U*, *L*, *S*)
*E*^*U*^(*X*)	The option value of the project under all equity financing
${\bar{V}}^{L}\left(X\right)$	The option value of the project under long-term debt financing
${\bar{V}}^{S}\left(X\right)$	The option value of the project under short-term debt financing

## Investment analysis under different financial arrangements

### Benchmark: Under the all equity capital financial arrangement

In this section, to better analyze the investment decision of the private firm under long-term debt financing and short-term debt financing, a benchmark scenario that the private firm only use equity capital to fund the construction cost is investigated. Suppose the option value of equity is *E*^*U*^(*X*), where the superscript “U” stands for the all equity capital scenario. Then, following the argument of previous works [24–26], *E*^*U*^(*X*) satisfies the following ordinary derivative equation:

$$\begin{array}{c} rE^{U}\left(X\right)=\mu E_{X}^{U}\left(X\right)+\frac{1}{2}\sigma^{2}E_{XX}^{U}\left(X\right) \end{array}$$ (1)

Where $E_{X}^{U}\left(X\right)$ and $E_{XX}^{U}\left(X\right)$ mean the first and second derivation of *E*^*U*^(*X*) on *X*. At the investment boundary $X_{I}^{U}$, the subscript “I” stands for the investment, the equity options value *E*^*U*^(*X*) satisfies

$$\begin{array}{c} E^{U}\left(X_{I}^{U}\right)=\frac{1-\tau }{r-\mu }X_{I}^{U}-I \end{array}$$ (2)

Combining with the condition *E*^*U*^(*X* = 0) = 0, it can be derived that

$$\begin{array}{c} E^{U}\left(X\right)=\left(\frac{1-\tau }{r-\mu }X_{I}^{U}-I\right){\left(\frac{X}{X_{I}^{U}}\right)}^{\beta_{2}} \end{array}$$ (3)

Where *β*_2_ is the positive solution of the equation $\frac{1}{2}\sigma^{2}\beta \left(\beta -1\right)+\mu \beta -r=0$, and $\beta_{2}=\frac{1}{2}-\frac{\mu }{\sigma^{2}}+\sqrt{{\left(\frac{1}{2}-\frac{\mu }{\sigma^{2}}\right)}^{2}+\frac{2r}{\sigma^{2}}}$.

The private firm will choose a best investment time to maximize the equity option value, that is to say, there exists an optimal investment boundary $X_{I}^{U}$ which is determined by the following optimal problem:

$$\begin{array}{c} X_{I}^{U}=\text{arg}\;\underset{X_{I}^{U}}{\text{max}}\left(\left(\frac{1-\tau }{r-\mu }X_{I}^{U}-I\right){\left(\frac{X}{X_{I}^{U}}\right)}^{\beta_{2}}\right) \end{array}$$ (4)

It can be derived that

$$\begin{array}{c} X_{I}^{U}=\frac{\beta_{2}}{\beta_{2}-1}\frac{r-\mu }{1-\tau }I \end{array}$$ (5)

### Under long-term debt financial arrangement

In this section, if the private firm can gain access to long-term debt, the optimal investment policies and default behaviour of the firm will be investigated. Once a decision is made to invest in the infrastructure project, the private firm needs to go through two important stages: the construction period and the operating period. During the construction period, the private firm needs to take cost *I* to construct the project. Since the investment is irreversible and the potential cash flow of the project is uncertain, there is a project threshold value $X_{I}^{L}$, and only when the estimated project value is larger than this threshold value will the private firm invest in the project. During the operating period, the private firm operates the project and collects project revenue to repay coupon payments and recover the original investment. However, the realized cash flow may not be sufficient to cover coupon payments, which may lead the private firm to default since the private firm has limited liability in the project. There exists a project threshold value $X_{B}^{L}$; when the realized project value is lower than this threshold value, the private firm will default and exit the project. Once the private firm defaults, the ownership of the project will be transferred to the debt-holder, and the debt-holder will liquidate the project. Because there exists managerial or asset specificity in the infrastructure project, the transfer of assets may deteriorate the cash flow of the project. Suppose the loss value of the project during the asset transfer process equals $\left(1-\alpha \right)X_{B}^{L}$, where *α* captures the recovery rate of the project in default, which relies on the degree of re-deployability of the project: a more redeployable project has a larger value of *α*, thus smaller loss in default.

During the operating period, the option value of equity expressed as *E*^*L*^(*X*) satisfies the ordinary derivative equation (ODE):

$$\begin{array}{c} rE^{L}\left(X\right)=\mu XE_{X}^{L}\left(X\right)+\frac{1}{2}\sigma^{2}X^{2}E_{XX}^{L}\left(X\right)+\left(1-\tau \right)\left(X-C^{L}\right) \end{array}$$ (6)

Where $E_{X}^{L}\left(X\right)$ and $E_{XX}^{L}\left(X\right)$ mean the first and second derivation of *E*^*L*^(*X*) on *X*. Besides, it also satisfies the other two boundary conditions:

$$\begin{array}{c} E^{L}\left(X_{B}^{L}\right)=0 \end{array}$$ (7)

$$\begin{array}{c} \frac{\partial E^{L}\left(X\right)}{\partial X}{|}_{X=X_{B}^{L}}=0 \end{array}$$ (8)

Combined with these two boundary conditions (2) and (3), the solution to the ODE can be derived as follows,

$$\begin{array}{c} E^{L}\left(X\right)=\frac{1-\tau }{r-\mu }X-\frac{1-\tau }{r}C^{L}-\left(\frac{1-\tau }{r-\mu }X_{B}^{L}-\frac{1-\tau }{r}C^{L}\right){\left(\frac{X}{X_{B}^{L}}\right)}^{\beta_{1}} \end{array}$$ (9)

Where *β*_1_ is a solution of the equation $\frac{1}{2}\sigma^{2}x\left(x-1\right)+\mu x-r=0$, and $\beta_{1}=\frac{1}{2}-\frac{\mu }{\sigma^{2}}-\sqrt{{\left(\frac{1}{2}-\frac{\mu }{\sigma^{2}}\right)}^{2}+\frac{2r}{\sigma^{2}}}<0$. The analytical expression of the default boundary $X_{B}^{L}$ is as follows,

$$\begin{array}{c} X_{B}^{L}=\frac{\beta_{1}}{\beta_{1}-1}\frac{r-\mu }{r}C^{L} \end{array}$$ (10)

Also, the project value expressed as *V*^*L*^(*X*) satisfies the ODE:

$$\begin{array}{c} rV^{L}\left(X\right)=\mu XV_{X}^{L}\left(X\right)+\frac{1}{2}\sigma^{2}X^{2}V_{XX}^{L}\left(X\right)+\left(1-\tau \right)X+\tau C^{L} \end{array}$$ (11)

When the cash flow value reaches the default boundary, the project value satisfies:

$$\begin{array}{c} V^{L}\left(X_{B}^{L}\right)=\frac{1-\tau }{r-\mu }\alpha X_{B}^{L} \end{array}$$ (12)

Solving the ODE by combining with the boundary condition (7) can derive the analytic expression of the project value.

$$\begin{array}{c} V^{L}\left(X\right)=\frac{1-\tau }{r-\mu }X+\frac{\tau }{r}C^{L}-\left(\left(1-\alpha \right)\frac{1-\tau }{r-\mu }X_{B}^{L}+\frac{\tau }{r}C^{L}\right){\left(\frac{X}{X_{B}^{L}}\right)}^{\beta_{1}} \end{array}$$ (13)

The project value expression comprises three parts: the accumulated cash flow value ($\frac{1-\tau }{r-\mu }X$), plus the tax benefits ($\frac{\tau }{r}C^{L}$), and minus the potential default loss ($\left(\left(1-\alpha \right)\frac{1-\tau }{r-\mu }X_{B}^{L}+\frac{\tau }{r}C^{L}\right){\left(\frac{X}{X_{B}^{L}}\right)}^{\beta_{1}}$). While using debt capital can bring tax benefits to the project, it also increases the default boundary of the private firm. Therefore, the private firm needs to trade off the benefits and costs of using debt capital to determine an optimal capital structure. Suppose the optimal debt level is *C*^L*^, which is determined by the following optimal problem:

$$\begin{array}{c} C_{L}^{*}=\text{arg}\;\underset{C}{\text{max}}\;V(C^{L}) \end{array}$$ (14)

According to the first order condition, the analytical expression of *C*^L*^ is derived:

$$\begin{array}{c} C^{L*}=\frac{\beta_{1}-1}{\beta_{1}}\frac{r}{r-\mu }{\left(1-\beta_{1}-\frac{\beta_{1}}{\tau }\left(1-\alpha \right)\left(1-\tau \right)\right)}^{\frac{1}{\beta_{1}}}X \end{array}$$ (15)

Denoting $\Delta ={\left(1-\beta_{1}-\frac{\beta_{1}}{\tau }\left(1-\alpha \right)\left(1-\tau \right)\right)}^{\frac{1}{\beta_{1}}}$, then, $C^{L*}=\frac{\beta_{1}-1}{\beta_{1}}\frac{r}{r-\mu }\Delta X$. Under the optimal debt level, the project value and the default boundary value can be respectively expressed as:

$$\begin{array}{c} V^{L}\left(X\right)=\frac{1}{r-\mu }\left(1-\tau +\tau \Delta \right)X \end{array}$$ (16)

$$\begin{array}{c} X_{B}^{L}=\Delta X \end{array}$$ (17)

Suppose $Q=\frac{1}{r-\mu }\left(1-\tau +\tau \Delta \right)$, then *V*^*L*^(*X*) = *QX*.

During the construction period, the private firm has to take cost of *I* to build the project. At this time, the project does not generate any cash flow. Besides, the private firm needs to search long-term debt financing with searching cost *ϕ*_1_, and can obtain long-term debt financing with probability of *δ*_1_. If the private firm fails to get a long-term debt contract, the competition that exists in the PPP market will make the private firm lose the investment opportunity. Therefore, the project value before investment ${\bar{V}}^{L}\left(X\right)$ satisfies the following ODE:

$$\begin{array}{c} r{\bar{V}}^{L}\left(X\right)=\mu X{\bar{V}}_{X}^{L}\left(X\right)+\frac{1}{2}\sigma^{2}X^{2}{\bar{V}}_{XX}^{L}\left(X\right)-\left(1-\delta_{1}\right){\bar{V}}^{L}\left(X\right)+\delta_{1}\left(V^{L}\left(X\right)-I\right)-\phi_{1} \end{array}$$ (18)

In the right hand of this equation, the third term means that when the private sector cannot get the long-term debt contract, the investment opportunity will be lost; the fourth term means that when the private firm obtains the long-term debt contract, the private firm can invest in the project and gain benefits of *V*^*L*^(*X*) − *I*. When the cash flow value reaches the investment boundary $X_{I}^{L}$, the following value matching condition is satisfied:

$$\begin{array}{c} {\bar{V}}^{L}\left(X_{I}^{L}\right)=V^{L}\left(X_{I}^{L}\right)-I \end{array}$$ (19)

Combining with Eqs (12) and (13), and ${\bar{V}}^{L}\left(0\right)=0$, the analytic expression of the project value before investment can be derived as:

$$\begin{array}{c} \begin{array}{c} {\bar{V}}^{L}\left(X\right)=\frac{\delta_{1}QX}{1+r-\delta_{1}-\mu }-\frac{\delta_{1}I+\phi_{1}}{1+r-\delta_{1}}+(\frac{\left(1+r-2\delta_{1}-\mu \right)QX_{I}^{L}}{1+r-\delta_{1}-\mu }\\ +\frac{\left(2\delta_{1}-1-r\right)I+\phi_{1}}{1+r-\delta_{1}}){\left(\frac{X}{X_{I}^{L}}\right)}^{\xi_{2}} \end{array} \end{array}$$ (20)

Where *ξ*_2_ is the positive solution of the equation $\frac{1}{2}\sigma^{2}\xi \left(\xi -1\right)+\xi \mu -\left(r+1-\delta_{1}\right)=0$, so $\xi_{2}=\frac{1}{2}-\frac{\mu }{\sigma^{2}}+\sqrt{{\left(\frac{1}{2}-\frac{\mu }{\sigma^{2}}\right)}^{2}+\frac{2(1+r-\delta_{1})}{\sigma^{2}}}$. Besides, at the investment boundary, to maximize the project value, the following smooth passing condition needs to be satisfied:

$$\begin{array}{c} \frac{\partial {\bar{V}}^{L}\left(X\right)}{\partial X}{|}_{X=X_{I}^{L}}=\frac{\partial V^{L}\left(X\right)}{\partial X}{|}_{X=X_{I}^{L}} \end{array}$$ (21)

Based on the above condition, the analytic expression of the investment boundary $X_{I}^{L}$ can be derived:

$$\begin{array}{c} X_{I}^{L}=\frac{\xi_{2}}{\xi_{2}-1}\frac{1+r-\delta_{1}-\mu }{2\delta_{1}+\mu -r-1}\frac{\left(2\delta_{1}-r-1\right)I+\phi_{1}}{1+r-\delta_{1}}\frac{1}{Q} \end{array}$$ (22)

### Under short-term debt financial arrangement

Due to the scarceness of long-term debt financing in the financial market, and in order to raise enough capitals for the construction cost, the private firm can alternatively consider to obtain a short-term debt contract first and then conduct the refinancing. In this section, suppose the private firm can only access short-term debt financing, the investment behavior of the private firm will be investigated just like the above section.

During the operating period, the option value of equity denoted as *E*^*S*^(*X*) satisfies the following ODE:

$$\begin{array}{c} rE^{S}\left(X\right)=\mu XE_{X}^{S}\left(X\right)+\frac{1}{2}\sigma^{2}X^{2}E_{XX}^{S}\left(X\right)+\left(1-\rho \right)\left(1-\tau \right)\left(X-C^{S}\right)+\rho \left(0-E^{S}\left(X\right)\right) \end{array}$$ (23)

Where (1 − *ρ*)(1 − *τ*)(*X* − *C*^*S*^) + *ρ*(0 − *E*^*S*^(*X*)) captures the effect of refinancing risk on the equity value. Besides, just like above section, the equity value *E*^*S*^(*X*) needs to satisfy the following conditions:

$$\begin{array}{c} \underset{X\to \infty }{\text{lim}}\;\frac{E^{S}\left(X\right)}{X}<\infty \end{array}$$ (24)

$$\begin{array}{c} E^{S}\left(X_{B}^{S}\right)=0 \end{array}$$ (25)

$$\begin{array}{c} \frac{\partial E^{S}\left(X\right)}{\partial X}{|}_{X=X_{B}^{S}}=0 \end{array}$$ (26)

Where $X_{B}^{S}$ means the boundary value that makes the private firm default and exit the project. Based on these equations, the analytic expression of the equity value can be derived, as follows:

$$\begin{array}{c} \begin{array}{c} E^{S}\left(X\right)=\frac{\left(1-\tau )(1-\rho \right)}{r+\rho -\mu }X-\frac{\left(1-\tau )(1-\rho \right)}{r+\rho }C^{S}-(\frac{\left(1-\tau )(1-\rho \right)}{r+\rho -\mu }X_{B}^{S}-\\ \frac{\left(1-\tau )(1-\rho \right)}{r+\rho }C^{S}){\left(\frac{X}{X_{B}^{S}}\right)}^{\gamma_{1}} \end{array} \end{array}$$ (27)

Where *γ*_1_ is the negative solution of the equation $\frac{1}{2}\sigma^{2}\gamma \left(\gamma -1\right)+\mu \gamma -\left(r+\rho \right)=0$, and $\gamma_{1}=\frac{1}{2}-\frac{\mu }{\sigma^{2}}-\sqrt{{\left(\frac{1}{2}-\frac{\mu }{\sigma^{2}}\right)}^{2}+\frac{2(r+\rho )}{\sigma^{2}}}$. The analytic expression of the default boundary value $X_{B}^{S}$ can also be derived:

$$\begin{array}{c} X_{B}^{S}=\frac{\gamma_{1}}{\gamma_{1}-1}\frac{r+\rho -\mu }{r+\rho }C^{S} \end{array}$$ (28)

The project value expressed as *V*^*S*^(*X*) satisfies the following ODE:

$$\begin{array}{c} rV^{S}\left(X\right)=\mu XV_{X}^{S}\left(X\right)+\frac{1}{2}\sigma^{2}X^{2}V_{XX}^{S}\left(X\right)+\left(1-\rho \right)\left(\left(1-\tau \right)X+\tau C^{S}\right)+\rho \left(0-V^{S}\left(X\right)\right) \end{array}$$ (29)

Besides, the project value satisfies the value matching condition at the default boundary $X_{B}^{S}$, that is to say,

$$\begin{array}{c} V^{S}\left(X_{B}^{S}\right)=\frac{1-\tau }{r-\mu }\alpha X_{B}^{S} \end{array}$$ (30)

And the non-bubble condition:

$$\begin{array}{c} \underset{X\to \infty }{\text{lim}}\;\frac{V^{S}\left(X\right)}{X}<\infty \end{array}$$ (31)

Combining with Eqs (23), (24) and (25) can derive the analytic expression of the project value:

$$\begin{array}{c} \begin{array}{c} V^{S}\left(X\right)=\frac{\left(1-\rho )(1-\tau \right)}{r+\rho -\mu }X+\frac{(1-\rho )\tau }{r+\rho }C^{S}-((\frac{\left(1-\rho )(1-\tau \right)}{r+\rho -\mu }\\ -\frac{1-\tau }{r-\mu }\alpha )X_{B}^{S}+\frac{1-\rho }{r+\rho }\tau C^{S}){\left(\frac{X}{X_{B}^{S}}\right)}^{\gamma_{1}} \end{array} \end{array}$$ (32)

The private firm chooses the optimal debt level *C*^S*^ to maximize the project value:

$$\begin{array}{c} C^{S*}=\text{arg}\;\underset{C^{S}}{\text{max}}\;V^{S}\left(C^{S}\right) \end{array}$$ (33)

Solving this optimization problem can obtain the analytic expression of the optimal debt level *C*^S*^:

$$\begin{array}{c} C^{S*}=\frac{\gamma_{1}-1}{\gamma_{1}}\frac{r+\rho }{r+\rho -\mu }\Sigma X \end{array}$$ (34)

where $\Sigma ={\left(1-\gamma_{1}-\frac{\gamma_{1}\left(1-\tau \right)}{\tau }\left(1-\frac{r+\rho -\mu }{\left(r-\mu )(1-\rho \right)}\alpha \right)\right)}^{\frac{1}{\gamma_{1}}}$.

***Assumption 1***: $0<\rho <\frac{\left(r-\mu )(1-\alpha \right)}{r-\mu +\alpha }$

This assumption provides an effective domain for short-term debt financing arrangement, i.e. the refinancing risk probability should not be too large, otherwise, short-term financing arrangement will be unfeasible to the private firm.

By analyzing the expression of *C*^S*^, a proposition is proposed as follows:

***Proposition 1***: The optimal debt level *C*^S*^ under short term debt financing displays an U-shape relationship with the refinancing risk probability *ρ*.

This proposition manifests that it is not a simple linear relationship between the optimal debt level and the refinancing risk probability. The private firm needs to trade off the tax shield benefits and the potential loss caused by the refinancing risk to determine the optimal debt level. When the refinancing risk probability is very small, the private firm does not need to worry about the refinancing risk, thus it can use comparatively more debt capitals to obtain more tax shield benefits. With increasing refinancing risk probability, the potential loss caused by the refinancing risk is increased. To reduce the difficulty of rolling over the debt financing, the private firm should use less debt capitals in the project. However, when the refinancing risk probability increases to a certain level, whether the private firm can roll over the current debt financing becomes very uncertain. To reduce the equity investment loss, the private firm tends to use less equity capitals and more debt capitals. Therefore, the principle-agent problem between the debt holder and the private firm is more severe when the refinancing risk probability is relative large.

Under the optimal debt level, the default boundary value of the private sector satisfies:

$$\begin{array}{c} X_{B}^{S}=\Sigma X \end{array}$$ (35)

By analyzing the expression of $X_{B}^{S}$, a proposition is proposed as follows:

***Proposition 2***: Under short-term debt financing arrangement, the default boundary of the private firm $X_{B}^{S}$ decreases with the probability of obtaining the debt financing *δ*_1_ and *δ*_2_.

This proposition manifests that when the private firm faces the refinancing risk, a better debt market can keep the private firm to invest in the project longer. That is to say, with a larger probability to get the refinancing capital, the private firm will exit the project later.

By comparing the expression of $X_{B}^{L}$ and $X_{B}^{S}$ (the Eqs (17) and (35)), the following proposition is established.

***Proposition 3***: The default threshold under the short-term debt scenario is larger than that under the long-term debt scenario ($X_{B}^{S}\geq X_{B}^{L}$), and increases with the refinancing risk.

This result reflects that different financial arrangements will cause different default behaviour of the private firm: the private sector will default earlier under short-term financial arrangement than under long-term financial arrangement. The intuition is that the private firm needs to face extra refinancing loss under short-term debt financial arrangement, which leads the private firm to exit the project earlier. Previous studies suggested that using debt capital would promote the private sector to default earlier than purely using equity capital [13, 19]. This study contributes to this line of studies by indicating that the default behaviour of the private firm is even different under different debt financial arrangements.

Under the optimal debt level, the project value *V*^*S*^(*X*) can be expressed as:

$$\begin{array}{c} V^{S}\left(X\right)=\frac{1-\rho }{r+\rho -\mu }\left(1-\tau +\tau \Sigma \right)X \end{array}$$ (36)

Denote $P=\frac{1-\rho }{r+\rho -\mu }\left(1-\tau +\tau \Sigma \right)$, then *V*^*S*^(*X*) = *PX*.

Suppose the project value before investment is ${\bar{V}}^{S}\left(X\right)$, and just like the argument in the above section, the project value ${\bar{V}}^{S}\left(X\right)$ satisfies the following ODE:

$$\begin{array}{c} r{\bar{V}}^{S}\left(X\right)=\mu X{\bar{V}}_{X}^{S}\left(X\right)+\frac{1}{2}\sigma^{2}X^{2}{\bar{V}}_{XX}^{S}\left(X\right)-\left(1-\delta_{2}\right){\bar{V}}^{S}\left(X\right)+\delta_{2}V^{S}\left(X\right)-\delta_{2}I-\phi_{2} \end{array}$$ (37)

At the investment boundary $X_{I}^{S}$, the project value satisfies the value matching condition:

$$\begin{array}{c} {\bar{V}}^{S}\left(X_{I}^{S}\right)=V^{S}\left(X_{I}^{S}\right)-I \end{array}$$ (38)

Combining the Eqs (30) and (31) and ${\bar{V}}^{S}\left(0\right)=0$, the analytic expression of the project value before investment can be derived:

$$\begin{array}{c} \begin{array}{c} {\bar{V}}^{S}\left(X\right)=\frac{\delta_{2}PX}{1+r-\delta_{2}-\mu }-\frac{\delta_{2}I+\phi_{2}}{1+r-\delta_{2}}+(\frac{\left(1+r-2\delta_{2}-\mu \right)PX_{I}^{S}}{1+r-\delta_{2}-\mu }\\ +\frac{\left(2\delta_{2}-1-r\right)I+\phi_{2}}{1+r-\delta_{2}}){\left(\frac{X}{X_{I}^{S}}\right)}^{\zeta_{2}} \end{array} \end{array}$$ (39)

Where *ζ*_2_ is the positive solution of the equation $\frac{1}{2}\sigma^{2}\zeta \left(\zeta -1\right)+\zeta \mu -\left(r+1-\delta_{2}\right)=0$, so $\zeta_{2}=\frac{1}{2}-\frac{\mu }{\sigma^{2}}+\sqrt{{\left(\frac{1}{2}-\frac{\mu }{\sigma^{2}}\right)}^{2}+\frac{2(1+r-\delta_{2})}{\sigma^{2}}}$. Besides, at the investment boundary, the project value satisfies the following smooth passing condition:

$$\begin{array}{c} \frac{\partial {\bar{V}}^{S}\left(X\right)}{\partial X}{|}_{X=X_{I}^{S}}=\frac{\partial V^{S}\left(X\right)}{\partial X}{|}_{X=X_{I}^{S}} \end{array}$$ (40)

Based on the above condition, the analytic expression of the investment boundary $X_{I}^{S}$ can be derived:

$$\begin{array}{c} X_{I}^{S}=\frac{\zeta_{2}}{\zeta_{2}-1}\frac{1+r-\delta_{2}-\mu }{2\delta_{2}+\mu -r-1}\frac{\left(2\delta_{2}-r-1\right)I+\phi_{2}}{1+r-\delta_{2}}\frac{1}{P} \end{array}$$ (41)

## Model implication and discussion

In this section, the dependence of the analytical result on the main parameters of the model is illustrated numerically, and several model predictions are given out correspondingly. In particular, we focus on the impact of different financial arrangements on the capital structure, investment boundary and default boundary of the private firm. The values of the basic parameters are determined based on data from project finance for capital-incentive investments [27, 28]: *r* = 0.05 for the risk-free interest rate, *μ* = 0.02 for the risk neutral expected rate of return, *σ* = 0.2 for the volatility of cash flow, *τ* = 0.3 for the tax rate, *δ*_1_ = 0.8 for the probability of accessing the long-term debt capital, *δ*_2_ = 0.9 for the probability of accessing the short-term debt capital, and *a* = 1 to get rid of the influence of improved financial market or better project profitability.

### Impact of different financing arrangements on the default boundary of the private firm

**Fig 1:**
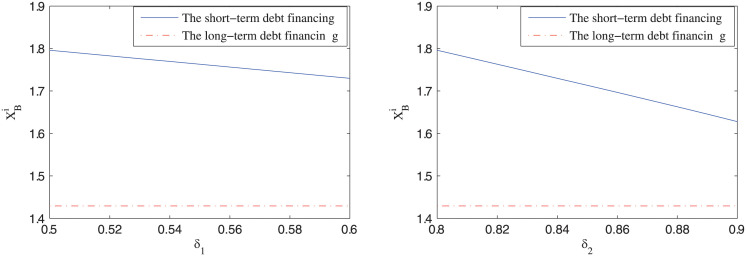
The impact of different financing arrangements on the default boundary of the private sector.

Fig 1 displays how the default boundary of the private firm $X_{B}^{i},\left(i=S,L\right)$ changes with respect to the probability of accessing debt capital *δ*_1_ and *δ*_2_, respectively. It can be seen that the default boundary of the private firm under short-term debt financing is larger than that under long-term debt financing, so the proposition 3 is numerically verified. Besides, it can be seen that the default boundary under short-term debt financing decreases with the probability of obtaining debt capital. These results reflect the influence of the financial market on the exit decision of the private firm in PPP projects, and can help explain why so many PPP projects ran into distress after the 2008 financial crises.

### Impact of different financing arrangements on the optimal capital structure of the private firm

**Fig 2:**
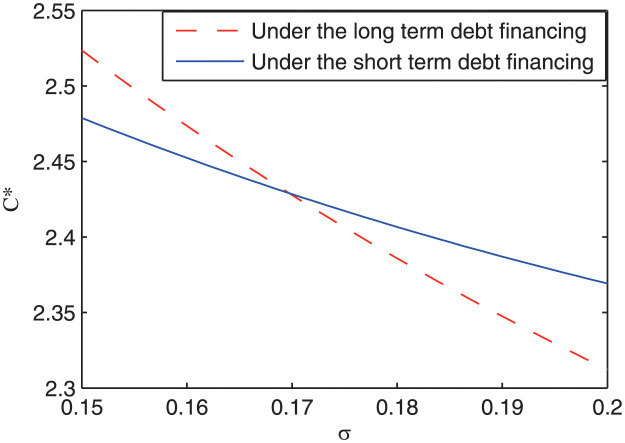
The impact of the volatility parameter on the capital structure of the private sector.

Fig 2 shows the optimal debt level C* with respect to the volatility of cash flow *σ*. It can be seen that both the optimal debt level under long-term debt financing and short-term debt financing decrease with the volatility of cash flow. The intuition is that a larger volatility of cash flow means more uncertainty about the benefits of the project. To reduce the potential default loss, the private firm should reduce the debt level of the project. Nonetheless, the optimal debt level under long-term debt financing decreases more quickly than that under short-term debt financing. When the volatility of cash flow is relatively small, the optimal debt level under long-term debt financing is larger than that under short-term debt financing. Therefore, there exists a volatility parameter value *σ*_0_; when the volatility of cash flow *σ* > *σ*_0_, the optimal debt level under short-term debt financing becomes larger than that under long-term debt financing. This result is an interesting new finding and reflects the difference in the optimal capital structure under different financial arrangements. The intuition is that compared to long-term debt financing, the private firm needs to deal with the refinancing risk under the short-term debt financing. When the volatility of cash flow is small, the cash flow of the project is relatively stable, and the private firm should raise relatively more debt capital to obtain more tax shield benefits. However, since the private firm needs to rollover the debt financing under short term debt financial arrangement, the optimal debt level under short-term debt financing should be lower than that under long-term debt financing. On the other hand, with the increase of the volatility of cash flow, the benefits of the project become more uncertainty, and the refinancing risk under short-debt financing will make the private sector unwilling to invest too much equity capital in the project. Therefore, the optimal debt level under short-term debt financing decreases relatively slower with the volatility of cash flow, and may be inversely larger than the optimal debt level under long-term debt financing. Based on the above observation, a prediction is proposed as follows:

***Prediction 1***: The optimal debt level under the long-term debt financial arrangement is larger than that under the short-term debt financial arrangement when the volatility of cash flow is small, while the reverse relationship is satisfied when the volatility of cash flow is large.

**Fig 3:**
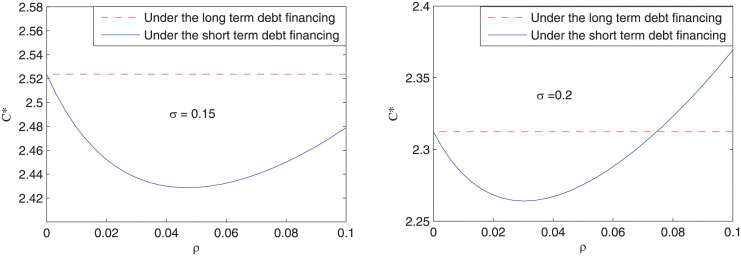
The impact of refinancing risk on the optimal debt level of the project.

Fig 3 depicts the impact of refinancing risk on the optimal debt level of the project. It can be seen that under short-term debt financing, there exists a U-shaped relationship between the optimal debt level C* and the probability of refinancing risk *ρ*. Besides, although the optimal debt level is decreased when the volatility of cash flow is increased from 0.15 to 0.2, the decreased debt level is larger when the refinancing risk probability is low than that when the refinancing risk probability is high. Numerically, C*(*ρ* = 0, *σ* = 0.15) − C*(*ρ* = 0, *σ* = 0.2) > C*(*ρ* = 0.1, *σ* = 0.15) − C*(*ρ* = 0.1, *σ* = 0.2). This result displays an asymmetric marginal influence of the volatility of cash flow on the optimal debt of the project, which constitutes the basis for the next predition:

***Prediction 2***: Under short-term debt financing, the difference in the optimal capital structure between projects with different volatility of cash flow is larger when the refinancing risk probability is low than that when the refinancing risk probability is high.

This result can help explain the degree of difference of the optimal capital structure in different projects: the higher the refinancing risk probability, the smaller is the degree of difference of the optimal capital structure in different projects. As far as our information goes, this is the first try to explore this question. Previous studies mainly focused on constructing a uniform framework to investigate the optimal capital structure, but could not explain why the difference of the optimal capital structure in different projects changes over time [14, 22, 29].

### Impact of different financing arrangements on the investment boundary of the private firm

**Fig 4:**
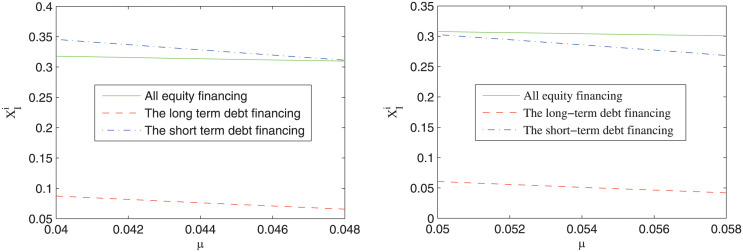
The impact of the risk neutral expect rate of return (*μ*) on the investment boundary.

Fig 4 shows the impact of the expected rate of return of the project on the investment boundary under different financial arrangements. It can be seen that with the increase of the expected rate of return of the project, the investment boundary of the private firm is decreased. That is to say, the more profitable the project, the earlier the private firm may invest in the project. Besides, it can also be seen that the investment boundary of the private firm is always the lowest under long-term debt financing, while the relative magnitude of the investment boundary under all equity financing and short-term debt financing relies on the value of the expected rate of return of the project. When the value of the expected rate of return of the project is relative small, the investment boundary of the private firm under all equity financing is lower than that under the short-term debt financing; on the other hand, when the value of the expected rate of return of the project is relative large, the investment boundary under the short-term debt financing is lower than that under all equity financing. This result manifests that the debt financing does not necessarily cause the so-called “over-investment problem” indicated in previous studies [30, 31]. When the project is not as profitable, short-term debt financing arrangement may deter the private firm from investing in the project, which supports the finding of previous studies that staged financing can help reduce the opportunistic behavior of the private firm [30, 31]. Based on the above analysis, a theoretical prediction is brought forward as follows.

***Prediction 3***: Among three financing arrangements, the private firm may invest in the project earliest under long-term debt financing. When the expected rate of return of the project is relative low, the private firm may invest in the project earlier under all equity financing than under short-term debt financing; otherwise, the reverse magnitude relationship is satisfied.

The prediction 3 provides a theoretical foundation for the government to afford TIFIA loan for the PPP project, since long-term debt financing can promote the private firm to invest in the PPP project earlier.

**Fig 5:**
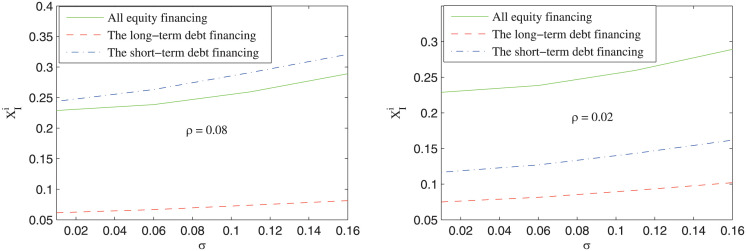
The impact of the volatility of cash flow (*σ*) on the investment boundary.

**Fig 6:**
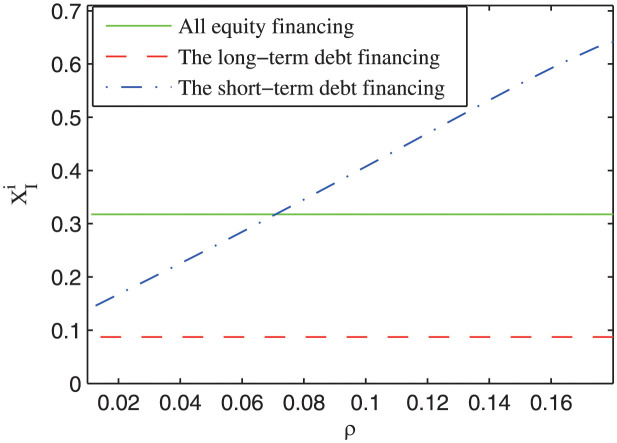
The impact of the refinancing risk (*ρ*) on the investment boundary.

Except the expected rate of return parameter, the volatility of cash flow and the refinancing risk parameters may also influence the investment boundary of the private firm. Fig 5 shows the impact of the volatility of cash flow on the investment boundary under different refinancing risk probabilities. It can be seen that the investment boundary increases with the volatility of cash flow. Namely, the more uncertain the cash flow of the project, the later the private sector may invest in the project. Besides, it can be also seen that the relative magnitude of the investment boundary depends on the value of the refinancing risk probability. When the refinancing risk probability is relatively large (*ρ* = 0.08), the private firm may invest earlier under all equity financing than under short-term debt financing; when the refinancing risk probability is relatively small (*ρ* = 0.02), the private firm may invest earlier under short-term debt financing than under the all equity financing. This result manifests that the refinancing risk is a critical factor that influences the investment decision of the private firm. As is shown in Fig 6, the investment boundary increases with the refinancing risk probability under short-term debt financing, which means that with larger refinancing risk probability, the private firm may invest in the project later. Based on the above analysis, a theoretical prediction is brought forward as follows.

***Prediction 4***: The more uncertain the cash flow of the project, the later the private firm may invest in the project. Besides, when the refinancing risk probability is relative low, the private firm may invest in the project earlier under short-term debt financing than under all equity financing; otherwise, the reverse magnitude relationship is satisfied.

**Fig 7:**
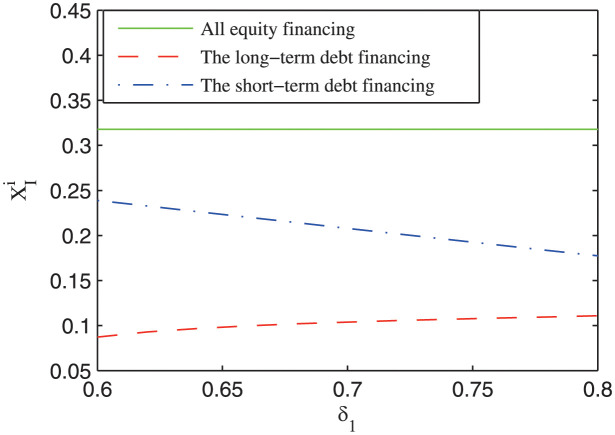
The impact of the probability of accessing long-term debt capitals (*δ*_1_) on the investment boundary.

Fig 7 depicts the impact of the probability of obtaining long-term debt financing on the investment boundary of the private firm. It can be seen that the investment boundary under short-term debt financing decreases with the probability of obtaining long-term debt financing, while the investment boundary under long-term debt financing increases with the probability of obtaining long-term debt financing. The intuition is that the private firm faces different financing status under different financial arrangements. Under long-tern debt financing, a high probability of obtaining long-term debt financing makes it more easy for the private firm to raise the needed financing, increasing the waiting option value of the private firm; hence the investment boundary of the private firm is increased and the private sector will invest in the project late. Under short-term debt financing, a higher probability of obtaining the long-term debt financing reduces the refinancing risk of the project, which promotes the private firm to invest in the project earlier. It is a new finding that the probability of obtaining long-term debt financing has a contrary influence on the investment boundary of the private firm under different financial arrangements, which can contribute to the literature about the investment decision analysis of the private firm [1, 23].

## Conclusion

This study investigates the influence of different financing arrangements on the investment behavior of the private firm. The result manifests that the investment behavior of the private firm is significantly different under different financing arrangements. First, the investment boundary of the private firm is the lowest under long-term debt financing, and the relative magnitude of the investment boundary under all equity financing and short-term debt financing relies on the value of the refinancing risk probability and the expected rate of return of the project. Second, the optimal debt level under long-term debt financing is larger than that under short-term debt financing when the volatility of cash flow is small, while the reverse relationship is satisfied when the volatility of cash flow is large. Besides, under short-term debt financing, the difference in the optimal capital structure between projects with different volatility of cash flow is larger when the refinancing risk probability is lower. Third, the optimal debt level under short-term debt financing displays a U-shaped relationship with the refinancing risk probability. Fourth, the private firm may exit the project earlier under short-term debt financing than under long-term debt financing. Finally, the probability of obtaining long-term debt financing has a contrary influence on the investment boundary of the private firm under different financial arrangements. These results can support the private firm to make better investment decision under different financing arrangements.

Some extensions of the model would be interesting. First, there may be some constraints for the private firm to raise debt financing from the bank, e.g. debt issuance limits [21]. These constraints can be included in the current framework to obtain more realistic results. Second, the private firm may both raise long-term and short-term debt financing, the optimal combination ratio of long-term debt financing and short-term debt financing can be further investigated. Third, private firms in the poject may have different interests that may make their investment decision inconsistency, how this inconsistency influence the investment decision of the project company should be further investigated.

## Supporting information

S1 Appendix (PDF)
