# Supplementary material for: Investment analysis of the private firm under different financial arrangements in infrastructure projects
Source: PLoS One. 2024 Feb 16;19(2):e0287418. doi: 10.1371/journal.pone.0287418 (PMC10871491; doi:10.1371/journal.pone.0287418)
Supplement: S1 Appendix — (PDF) [file pone.0287418.s001.pdf]

S1 Appendix. Proof of Proposition 1:

Denote $f_1(\rho) = \frac{\gamma_1 - 1}{\gamma_1}\frac{r+\rho}{r+\rho-\mu}$, and $f_2(\rho) = \Sigma$, then $C^{S*}(\rho) = f_1(\rho)f_2(\rho)X$,
$C^{S*''}(\rho) = (f_1''(\rho)f_2(\rho) + 2f_1'(\rho)f_2'(\rho) + f_1(\rho)f_2''(\rho))X$.
$lnf_1(\rho) = ln(\gamma_1 - 1) - ln\gamma_1 + ln(r+\rho) - ln(r+\rho-\mu)$, so
$f_1^{'}(\rho) = (\frac{d\gamma_1}{d\rho}\frac{1}{\gamma_1(\gamma_1-1)} - \frac{\mu}{(r+\rho)(r+\rho-\mu)})f_1(\rho) < 0$,

$f_1''(\rho) = (\frac{d^2\gamma_1}{d\rho^2}\frac{1}{\gamma_1(\gamma_1-1)} - (\frac{d\gamma_1}{d\rho})^2\frac{2\gamma_1-1}{(\gamma_1(\gamma_1-1))^2} + (\frac{d\gamma_1}{d\rho}\frac{1}{\gamma_1(\gamma_1-1)} - \frac{\mu}{(r+\rho)(r+\rho-\mu)})^2)f_1(\rho) > 0$.

$lnf_2(\rho) = \frac{ln(1-\gamma_1 - \frac{\gamma_1(1-\tau)}{\tau}(1-\frac{(r+\rho-\mu)\alpha}{(r-\mu)(1-\rho)}))}{\gamma_1}$. According to Taylor expansion rule,
$lnf_2(\rho) = -(1 + \frac{1-\tau}{\tau}(1 - \frac{r+\rho-\mu}{(r-\mu)(1-\rho)}\alpha))$, so $f_2^{'}(\rho) = \frac{1-\tau}{\tau}\frac{r+1-\mu}{(1-\rho)^2}\alpha f_2(\rho) > 0$,
$f_2^{''}(\rho) = (2\frac{1-\tau}{\tau}\frac{r-\mu+1}{(1-\rho)^3}\alpha + (\frac{1-\tau}{\tau}\frac{r+1-\mu}{(1-\rho)^2}\alpha)^2)f_2(\rho) > 0$.
$C^{S*''}(\rho) = (f_1''(\rho) + 2\frac{1-\tau}{\tau}\frac{r+1-\mu}{(1-\rho)^2}\alpha f_1'(\rho))f_2(\rho) + f_1(\rho)f_2''(\rho))X$. When the value of ρ is relative small, $f_1''(\rho) + 2\frac{1-\tau}{\tau}\frac{r+1-\mu}{(1-\rho)^2}\alpha f_1'(\rho) > 0$, so $C^{S*''}(\rho) > 0$. The proposition 1 is proven.

Proof of Proposition 2:

It can be derived that $\frac{dX_B^S}{d\delta_1} = \frac{df_2(\delta_1)}{d\delta_1}X$. Since $\frac{df_2(\delta_1)}{d\delta_1} = \frac{df_2(\rho)}{d\rho}\frac{d\rho}{\delta_1}$, and $\frac{df_2(\rho)}{d\rho} > 0$, $\frac{d\rho}{\delta_1} < 0$, it can be seen that $\frac{dX_B^S}{d\delta_1} < 0$. In a similar way, it can be derived that $\frac{dX_B^S}{d\delta_2} < 0$. The proposition 2 is proven.

Proof of Proposition 3:

$X_B^L = \Delta X, X_B^S = \Sigma X$. It can be derived that when $\rho = 0$, $X_B^L = X_B^S$. In addition, duo to $\frac{dX_B^S}{d\rho} > 0$, it can be derived that $X_B^L < X_B^S$. The proposition 3 is proven.
